# Medical Institutions in Germany

**Published:** 1844-09-21

**Authors:** 


### RECORD OF MEDICAL SCIENCE.

### MEDICAL INSTITUTIONS IN GERMANY.

The most important feature of the medical institutions of the German states are:—First. The existence of various grades of practitioners. Second. The University honorary degrees. Third. The *staats-examen*, or examination for the license to practice. Fourth. The existence of a medical hierarchy, composed of public medical functionaries, paid by government.

In no part of Europe is the medical profession more divided and subdivided than in Germany. Not only are there practitioners of a superior and inferior rank, but those who belong to the latter are subdivided into two, three, even four grades. It is a remarkable fact, that in Germany, as elsewhere, it is practitioners of surgery who occupy the lower ranks of the profession. Thus we have surgeons of the first, second, and third class, and assistants to the surgeons. They who practise medicine, on the contrary, are placed in the higher rank, and are always, or nearly always, doctors and graduates of of an university. This fact, which at first appears singular, is, however, easily accounted for, if we refer to the history of the healing art. The distinction between surgery and medicine certainly did not exist until the middle ages. In the days of GALEN there were, it is true, in Rome, some who practised surgery nearly exclusively, but it was from taste, not because the separation between surgery and medicine was generally recognised by the profession. They devoted themselves to surgery in the same way as many others devoted themselves to other specialities, such as diseases of the eye, of the ear, fractures, luxations, &c. In the middle ages, however, the study and practice of medicine, as well as of all other sciences, fell into the hands of the clergy; and as their religious tenets forbade the shedding of blood, they confined themselves to the duties of the physician, leaving all operations to be performed by subordinates, barber-surgeons, who operated under their guidance. Thence it is that surgery fell into a degree of disrepute and contempt from which it has not even yet recovered; and that although the state of things in which such disrepute originated has long since ceased to exist. Indeed, it would be difficult to find a more flagrant instance of the tenacity with which bodies of men cling to antiquated prejudices, even when every shadow of reason for such conduct has departed, than in the position which surgery still occupies. For centuries the public has ceased, in England, at least, to attach any idea of degradation to the performance of surgical operations, and yet we still find our surgeons voluntarily placed on a lower social footing than physicians, expelled from our universities, and considered unworthy of the title (that of doctor) which has, since the revival of learning, been granted to those who attain proficiency in any scientific pursuits. We say voluntarily, because there has been nothing in the conduct of our surgeons, as a body, to indicate that they consider themselves aggrieved by the position which they still occupy in society.

In Germany there are doctors in surgery, and the degree of doctor is generally taken by those who, having received a good education, intend to practise as surgeons. The doctors in surgery are, however, principally confined to the large towns, and in smaller localities the practice of surgery is thrown into the hands of the doctors in medicine and surgery (general practitioners) and of the subordinate surgeons; the latter, generally speaking, also practise medicine, although they do so illegally. The subordinate practitioners are, mostly, men of little or no education, who originate from the lower ranks of the middle classes. Their wants being, consequently, few, they are satisfied with a very trifling remuneration, and thus undermine the regularly-educated doctors. The result of this competition between the different grades of medical practitioners is that the scale of remuneration has been reduced to so low a point as to be totally inadequate to compensate a well-educated man for his time and labour. Owing to this circumstance, and to the degradation which has been thus entailed upon the medical profession, great efforts have been made for many years, by the graduated practitioners, to induce Government to abolish all subordinate classes. The subject has, of late, attracted much attention, and is now under discussion. It is generally believed that the Prussian Government will shortly consent to suppress the inferior grades of practitioners, confining the exercise of the profession to those who have passed through the university curriculum and taken the degree of doctor.

The title of doctor, in every part of Germany, with the exception of Bavaria, is merely honorary. It does not confer a right to practise, and is no guarantee whatever of medical knowledge being granted after a merely nominal examination. The license to practise, however, is only obtained, as we have stated, by submitting to a severe examination before a special board (*staats-examen*). This is a most unfortunate regulation, as it tends to lessen the dignity of the medical profession, by crowding it with men who have no pretensions to medical knowledge. It also explains the facility with which the diploma of M. D. is granted to foreigners by many German universities, and proves how worthless such diplomas really are in a scientific point of view.

The *staats-examen* is a necessary safeguard in a country where the medical degree has no medical value. It appears to be carried on in such a manner as to be a tolerable test of a candidate's knowledge. The entire series of studies and ordeals, however, through which a student has to pass before he obtains the right to practise, is more calculated to make him a learned than a practical medical man. The clinical studies of the student are, indeed, everywhere exceedingly defective.

A very remarkable feature in the medical organisation of Germany is the existence of the public medical functionaries, who are entrusted with a species of jurisdiction over the medical profession in general, and constitute, as it were, a medical police. They have existed in Germany from a very remote period, and the institution everywhere remains unaltered, whatever other reforms are effected. This circumstance seems to prove that it is found by the different governments to be of real value. Indeed, it is evident that a body of medical functionaries, whose attention is particularly directed to questions relating to public hygiene and to legal medicine, must prove of great service to the German governments, which always show a deep interest in the physical welfare of the population intrusted to their care. By their union they form, as it were, boards of health, the advice and co-operation of which can always be easily obtained by the local authorities. They also exercise a wholesome restraint on the medical pratitioners over whom they preside. In this respect we ourselves are deplorably deficient, and it would be well were our Government to establish some substitute for the German medical hierarchy and the French Boards of Health. We cannot, however, but strongly disapprove of the conduct of some of the German states in turning the medical functionaries into spies on their professional brethren, and thus making a medical institution subservient to political purposes.

In Bavaria very great changes have been recently enacted. The examinations for the medical degree have been constituted the real test of a student's medical knowledge, a great improvement on the system followed elsewhere. Consequently the *staats-examen* has been dispensed with. The inferior practitioners have been all but abolished, and the number of practitioners in each locality has been limited. We are inclined to think that by this last enactment the Bavarian reformers have gone rather too far. We very much doubt whether it be really judicious to limit, by authority, the number of practitioners in a given district. This latter regulation has given great dissatisfaction in Bavaria, and principally because it has been made an instrument of tyranny in the hands of the rulers, the vacant appointments being only given to those whose political and religious principles are approved of.

On the whole the medical organisation of Germany is at present, in many respects, very defective, such, indeed, as to conduce but little to the improvement of medicine as a science, and to insure but little dignity or profit to those who practise it. Defective as the state of the medical profession is in Great Britain, there is certainly but little we can envy our German brethren.—*London Lancet.*

